# Supplementary material for: ABHD11 inhibition drives sterol metabolism to modulate T cell effector function and alleviate autoimmunity
Source: bioRxiv. 2025 Mar 19:2025.03.19.643996. Preprint. [Version 1] doi: 10.1101/2025.03.19.643996 (PMC11957007; doi:10.1101/2025.03.19.643996)
Supplement: 1 [file NIHPP2025.03.19.643996V1-supplement-1.pdf]

bioRxiv preprint doi: https://doi.org/10.1101/2025.03.19.643996; this version posted March 19, 2025. The copyright holder for this preprint (which was not certified by peer review) is the author/funder, who has granted bioRxiv a license to display the preprint in perpetuity. It is made available under aCC-BY 4.0 International license.

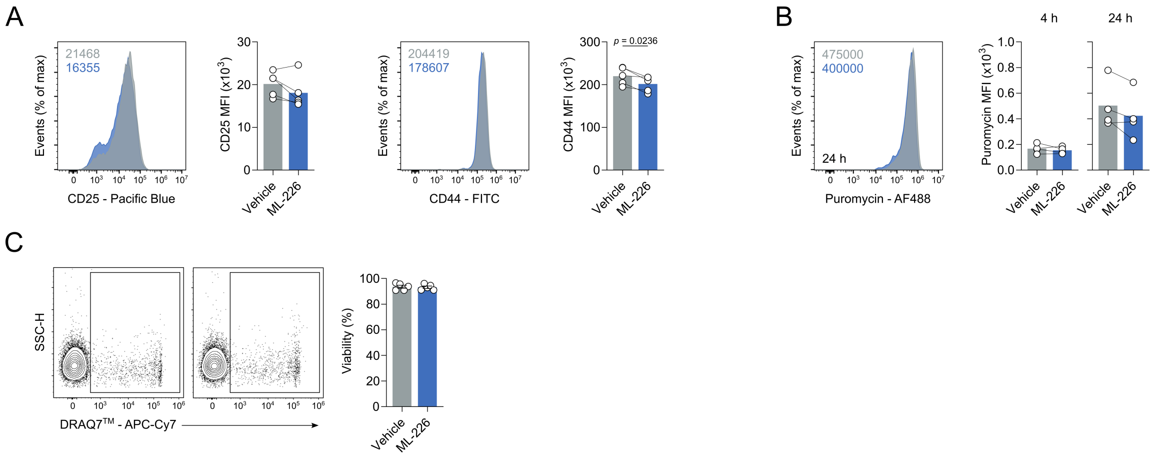

Supplementary Figure 1. AHBD11 inhibition does not significantly alter T cell size, protein translation and viability

(**A**) Surface expression of activation markers (CD25 and CD44), as measured by flow cytometry, on CD4+ effector T cells (n = 5). (**B**) Puromycin incorporation, as measured by flow cytometry, in CD4+ effector T cells (n = 4). (**C**) Cell viability, as determined by DRAQ7®, in CD4+ effector T cells (n = 5). All experiments were carried out using human samples. CD4+ T cells were activated with α-CD3 and α-CD28 for 24 h, in the presence and absence of ML-226, unless otherwise stated. Data are expressed as either: mean, with paired dots representing biological replicates; or mean ± SEM.

bioRxiv preprint doi: https://doi.org/10.1101/2025.03.19.643996; this version posted March 19, 2025. The copyright holder for this preprint (which was not certified by peer review) is the author/funder, who has granted bioRxiv a license to display the preprint in perpetuity. It is made available under aCC-BY 4.0 International license.

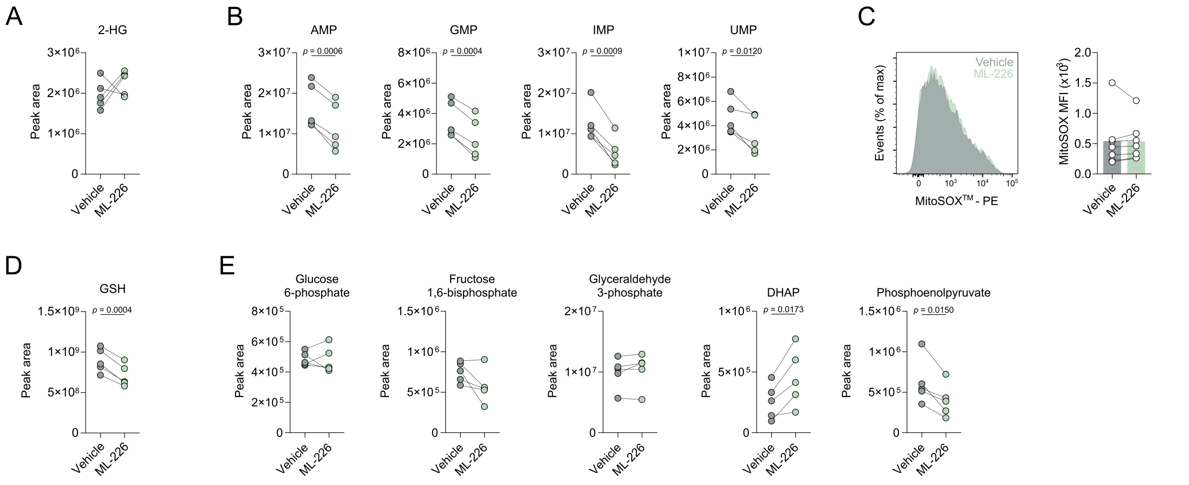

Supplementary Figure 2. ABHD11 inhibition reduces intracellular monophosphate nucleotides

(**A**) Intracellular levels of 2-hydroxyglutarate (2-HG) in CD4+ T cells (n = 5). (**B**) Intracellular levels of selected monophosphate nucleotides in CD4+ effector T cells activated with α-CD3 and α-CD28 for 24 h, in the presence and absence of ML-226 (n = 5). Metabolites include: inosine monophosphate, adenosine monophosphate, guanosine monophosphate and uridine monophosphate. (**C**) Mitochondrial ROS levels, as determined by MitoSOX™ Red, in CD4+ effector T cells (n = 7). (**D**) Intracellular levels of glutathione in CD4+ effector T cells (n = 5). (**E**) Intracellular levels of selected glycolytic intermediates in CD4+ effector T cells (n = 5). Metabolites include: glucose 6-phosphate, fructose 1,6-bisphosphate, glyceraldehyde 3-phosphate, dihydroxyacetone phosphate (DHAP) and phosphoenolpyruvate. All experiments were carried out using human samples. CD4+ T cells were activated with α-CD3 and α-CD28 for 24 h, in the presence and absence of ML-226, unless otherwise stated. Data are expressed as mean, with paired dots representing biological replicates.

bioRxiv preprint doi: https://doi.org/10.1101/2025.03.19.643996; this version posted March 19, 2025. The copyright holder for this preprint (which was not certified by peer review) is the author/funder, who has granted bioRxiv a license to display the preprint in perpetuity. It is made available under aCC-BY 4.0 International license.

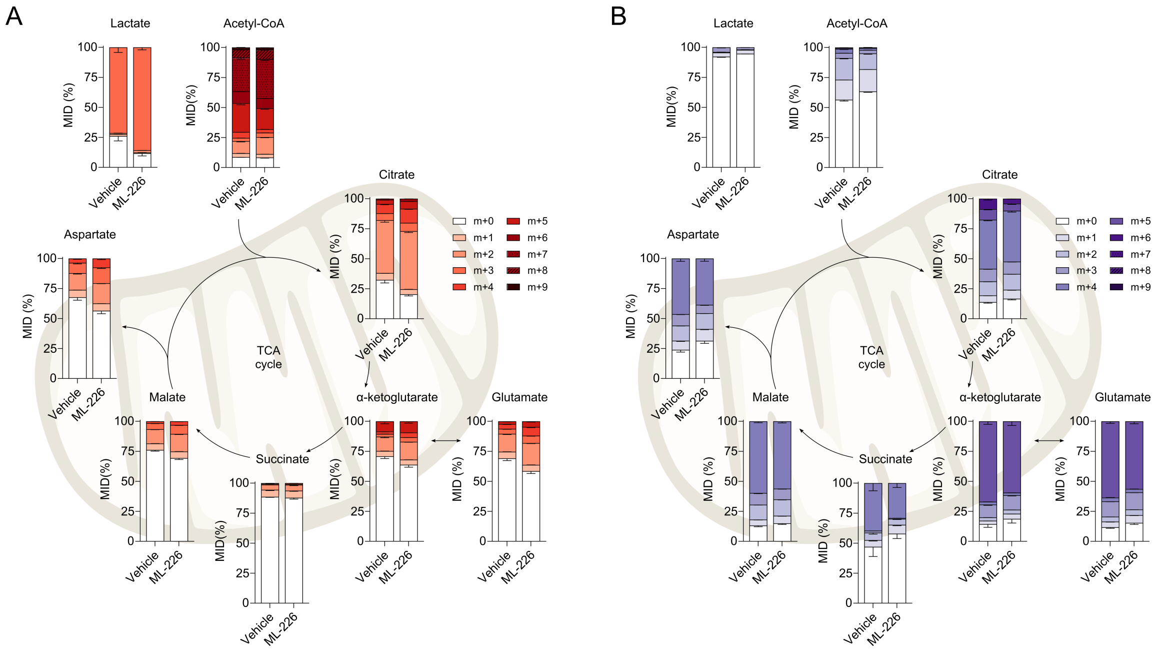

Supplementary Figure 3. ABHD11 inhibition alters glucose and glutamine utilisation
(**A**) Stable isotope tracing of uniformly labelled $^{13}C_6$-glucose into the TCA cycle and related intermediates in CD4+ effector T cells (n = 6). (**B**) Stable isotope tracing of uniformly labelled $^{13}C_5$-glutamine into the TCA cycle and related intermediates in CD4+ effector T cells (n = 6). Metabolites include: lactate, acetyl-CoA, citrate, α-ketoglutarate, glutamate, succinate, malate and aspartate. Mass isotopologue distribution (MID) represented as the proportion of the metabolite pool. All experiments were carried out using human samples. CD4+ T cells were activated with α-CD3 and α-CD28 for 24 h, in the presence and absence of ML-226, unless otherwise stated. Data are expressed as mean ± SEM.

bioRxiv preprint doi: https://doi.org/10.1101/2025.03.19.643996; this version posted March 19, 2025. The copyright holder for this preprint (which was not certified by peer review) is the author/funder, who has granted bioRxiv a license to display the preprint in perpetuity. It is made available under aCC-BY 4.0 International license.

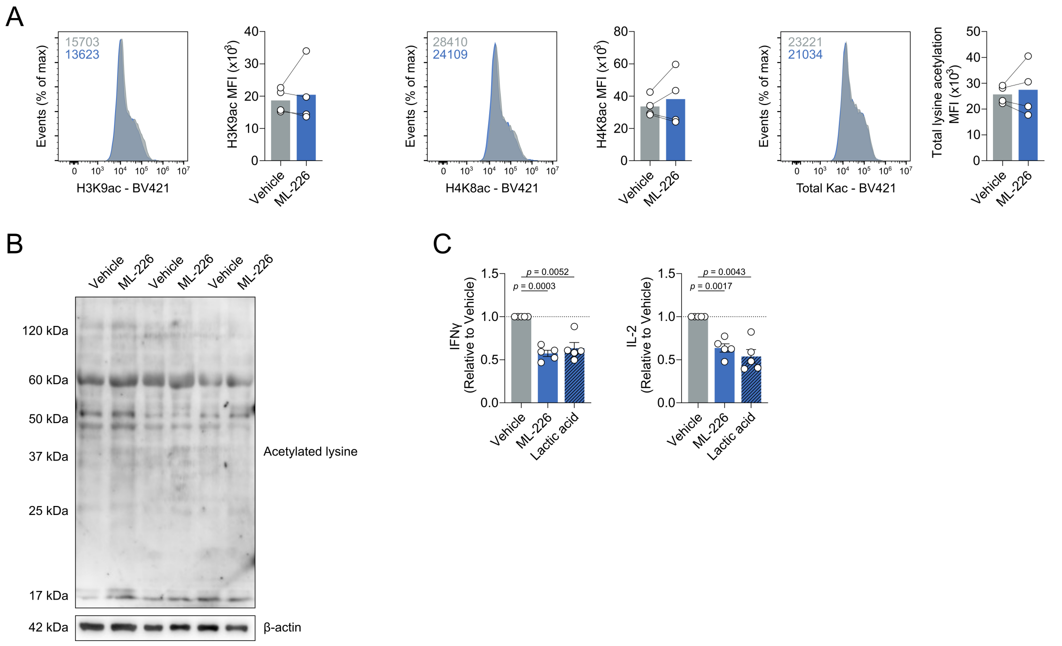

Supplementary Figure 4. ABHD11 inhibition has contrasting effects on epigenetic modifications

(**A**) Intracellular histone acetylation levels, as measured by flow cytometry, in CD4+ effector T cells (n = 4). Histone acetylation measured on: H3K9 and H4K8. (**B**) Total lysine acetylation in CD4+ T cells (n = 3). Protein loading assessed using β-actin. (**C**) IL-2 and IFNγ production by CD4+ T cells, activated in the presence and absence of ML-226 or lactic acid (n = 5). All experiments were carried out using human samples. CD4+ T cells were activated with α-CD3 and α-CD28 for 24 h. Data are expressed as either: mean, with paired dots representing biological replicates; or mean ± SEM.

1224
1225

bioRxiv preprint doi: https://doi.org/10.1101/2025.03.19.643996; this version posted March 19, 2025. The copyright holder for this preprint (which was not certified by peer review) is the author/funder, who has granted bioRxiv a license to display the preprint in perpetuity. It is made available under aCC-BY 4.0 International license.

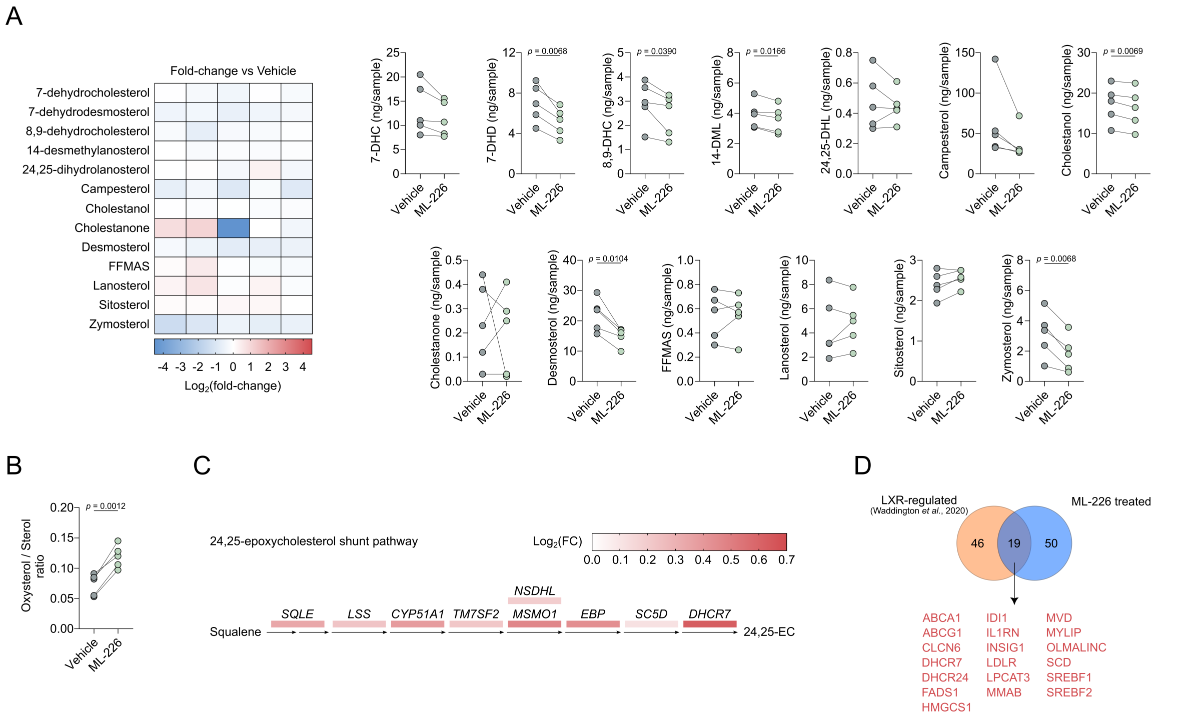

Supplementary Figure 5. ABHD11 inhibition reduces non-oxygenated sterol levels and drives activation of a mevalonate shunt pathway

(**A**) Intracellular levels of selected non-oxygenated sterols in CD4+ T cells (n = 5). Metabolites include: 7-dehydrocholesterol, 7-dehydrodesmosterol, 8,9-dehydrocholesterol, 14-desmethylanosterol, 24,25-dihydrolanosterol, campesterol, cholestanol, cholestanone, desmosterol, follicular fluid meiosis-activating sterol (FFMAS), lanosterol, sitosterol, zymosterol. Heatmap represented as Log_2(fold-change) versus vehicle control. (**B**) Oxysterol / sterol ratio in CD4+ T cells (n = 5). (**C**) Changes in enzyme transcript levels within the 24,25-epoxycholesterol shunt pathway, as measured by RNA-seq, in CD4+ T cells (n = 4). (**D**) Overlap between liver X receptor-associated genes and genes differentially-regulated by ABHD11 inhibition in CD4+ T cells (n = 4). All experiments were carried out using human samples. CD4+ T cells were activated with α-CD3 and α-CD28 for 24 h, in the presence and absence of ML-226. Data are expressed as mean, with paired dots representing biological replicates.

bioRxiv preprint doi: https://doi.org/10.1101/2025.03.19.643996; this version posted March 19, 2025. The copyright holder for this preprint (which was not certified by peer review) is the author/funder, who has granted bioRxiv a license to display the preprint in perpetuity. It is made available under aCC-BY 4.0 International license.

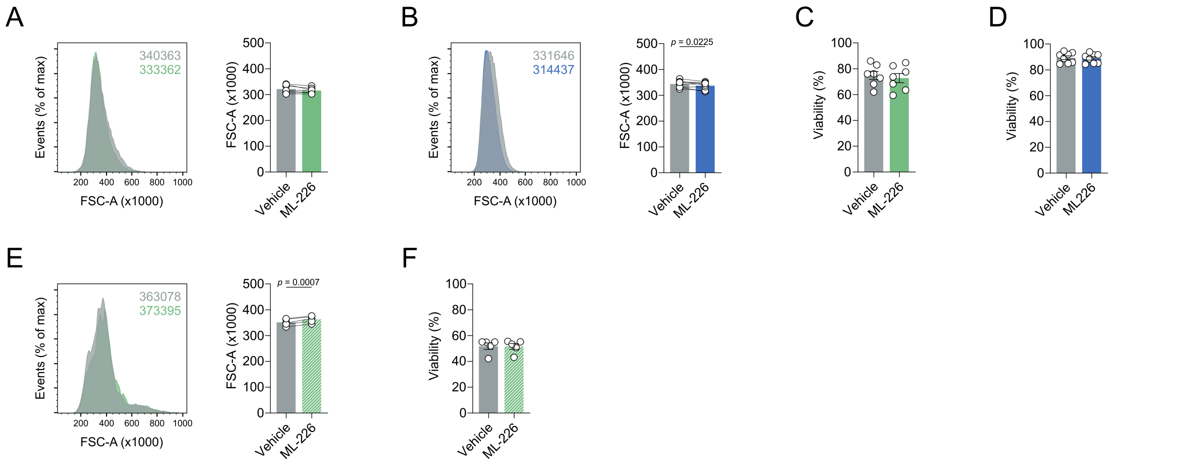

Supplementary Figure 6. ABHD11 inhibition has no clear effect on T cell size and viability in autoimmune patient cohorts

(**A,B**) Cell size, as determined by forward scatter area, of patient-derived CD4+ T cells in autoimmune cohorts of (**A**) RA (n = 7) and (**B**) T1D (n = 8). (**C,D**) Cell viability, as determined by DRAQ7®, in patient-derived CD4+ T cells in autoimmune cohorts of (**C**) RA (n = 7) and (**D**) T1D (n = 8). (**E**) Cell size, as determined by forward scatter area, of patient-derived synovial fluid mononuclear cells (SFMCs; n = 5). (**F**) Cell viability, as determined by DRAQ7®, in patient-derived SFMCs (n = 5). All experiments were carried out using human samples. CD4+ T cells were activated with α-CD3 and α-CD28 for 24 h, in the presence and absence of ML-226, unless otherwise stated. Data are expressed as either: mean, with paired dots representing biological replicates; or mean ± SEM.

bioRxiv preprint doi: https://doi.org/10.1101/2025.03.19.643996; this version posted March 19, 2025. The copyright holder for this preprint (which was not certified by peer review) is the author/funder, who has granted bioRxiv a license to display the preprint in perpetuity. It is made available under aCC-BY 4.0 International license.

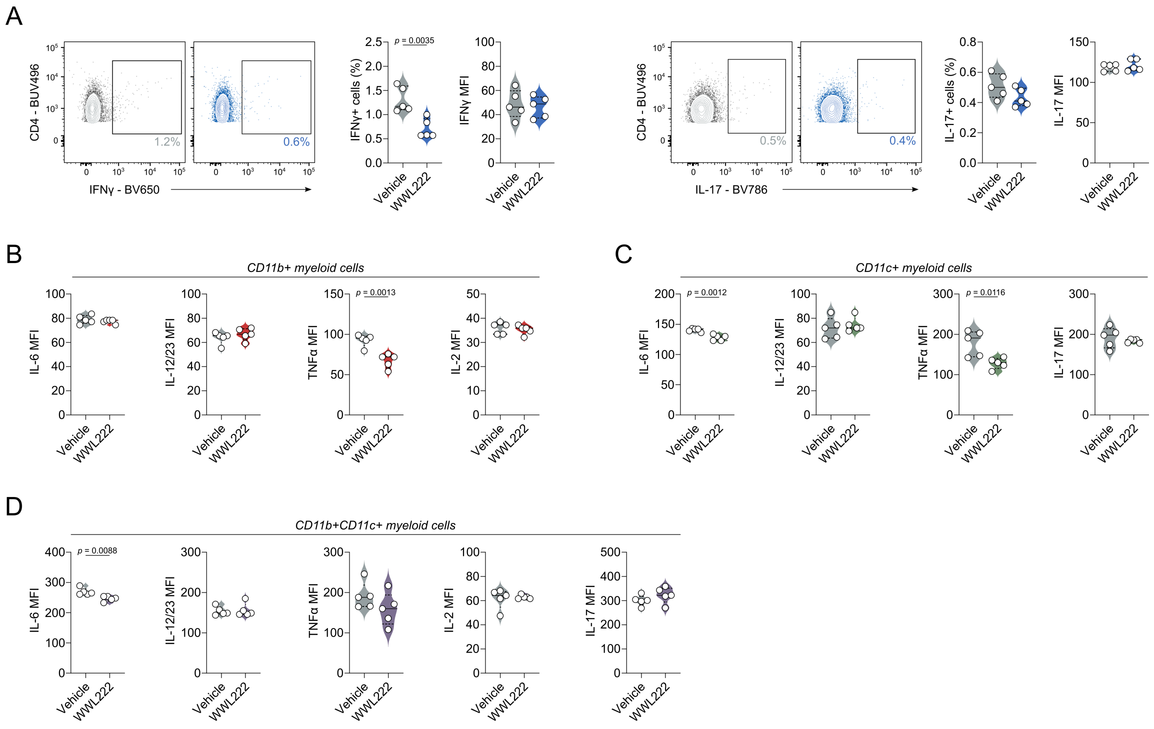

Supplementary Figure 7. ABHD11 inhibition delays T1D by altering the cytokine profile

(**A**) IFNγ and IL-17 production, as measured by flow cytometry, by CD4+ T cells (n = 5). (**B**) IL-6, IL-12/23, TNFα and IL-2 production, as measured by flow cytometry, by splenic CD11b^{+} myeloid cells (n = 5). (**C**) IL-6, IL-12/23, TNFα and IL-17 production, as measured by flow cytometry, by splenic CD11c^{+} myeloid cells (n = 5). (**D**) IL-6, IL-12/23, TNFα, IL-2 and IL-17 production, as measured by flow cytometry, by splenic CD11b^{+}CD11c^{+} myeloid cells (n = 5). All experiments were carried out using murine samples. Mice were injected daily with the indicated dose of WWL222. Data are expressed as median ± interquartile range.

bioRxiv preprint doi: https://doi.org/10.1101/2025.03.19.643996; this version posted March 19, 2025. The copyright holder for this preprint (which was not certified by peer review) is the author/funder, who has granted bioRxiv a license to display the preprint in perpetuity. It is made available under aCC-BY 4.0 International license.

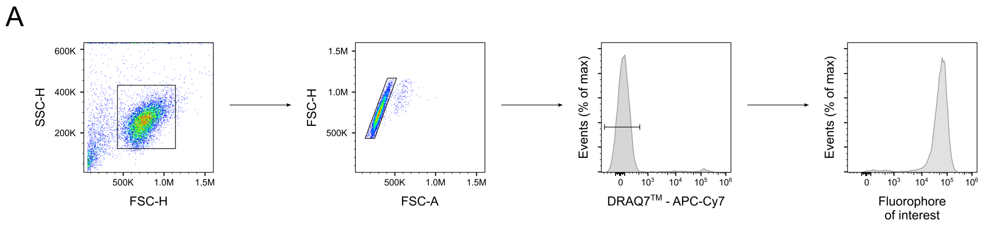

Supplementary Figure 8. Representative gating strategy

(**A**) Representative gating strategy employed for flow cytometry analysis. Cell doublets were excluded from analysis based on forward scatter-height versus forward scatter-area. Cell death was monitored using DRAQ7™ (1 µM; Biostatus, UK) and dead cells were excluded from analysis.

bioRxiv preprint doi: https://doi.org/10.1101/2025.03.19.643996; this version posted March 19, 2025. The copyright holder for this preprint (which was not certified by peer review) is the author/funder, who has granted bioRxiv a license to display the preprint in perpetuity. It is made available under aCC-BY 4.0 International license.

Supplementary Table 1. Rheumatoid arthritis patient demographics

	Rheumatoid arthritis	
**Patient demographics**	**PBMCs (n = 7)**	**SFMCs (n = 5)**
**Age**		
Mean (SD)	68.6 (± 6.45)	66.0 (± 5.05)
Median	68.0	66.0
Range	57.0 – 77.0	60.0 – 71.0
**Sex** n (%)		
Female	5 (71.4%)	3 (60.0%)
Male	2 (28.6%)	2 (40.0%)
Not determined	0 (0.0%)	0 (0.0%)
**Treatment**		
No medication	1 (14.2%)	2 (40.0%)
bDMARD only	4 (57.1%)	1 (20.0%)
csDMARD only	2 (28.6%)	1 (20.0%)
bDMARD + csDMARD	0 (0.0%)	0 (0.0%)
bDMARD + TNF inhibitor	0 (0.0%)	0 (0.0%)
Not determined	0 (0.0%)	1 (20.0%)
**Rheumatoid Factor**		
Positive	4 (57.1%)	0 (0.0%)
Negative	3 (42.9%)	0 (0.0%)
Not determined	0 (0.0%)	5 (100.0%)
**ACPA**		
Positive	4 (57.1%)	0 (0.0%)
Negative	3 (42.9%)	0 (0.0%)
Not determined	0 (0.0%)	5 (100.0%)
**DAS28**		
Mean (SD)	4.59 (± 2.57)	3.56 (± 0.30)
Median	3.84	3.64
Range	2.47 – 7.60	3.13 – 3.83

ACPA, anti-citrullinated protein antibodies; DAS28, disease activity score; DMARD, disease-modifying antirheumatic drugs; PBMCs, peripheral blood mononuclear cells; SFMCs, synovial fluid mononuclear cells

bioRxiv preprint doi: https://doi.org/10.1101/2025.03.19.643996; this version posted March 19, 2025. The copyright holder for this preprint (which was not certified by peer review) is the author/funder, who has granted bioRxiv a license to display the preprint in perpetuity. It is made available under aCC-BY 4.0 International license.

Supplementary Table 2. Type 1 diabetes patient demographics.

Patient demographics	Type 1 diabetes (n = 8)
**Age**	
Mean (SD)	36.4 (± 14.3)
Median	32.4
Range	19.0 – 62.0
**Sex** n (%)	
Female	7 (87.5%)
Male	1 (12.5%)
**Diabetes duration**	
Mean (SD)	14.2 (± 14.8)
Median	11.06
Range	1.8 – 47.0
**Treatment**	
Insulin	8 (100.0%)

bioRxiv preprint doi: https://doi.org/10.1101/2025.03.19.643996; this version posted March 19, 2025. The copyright holder for this preprint (which was not certified by peer review) is the author/funder, who has granted bioRxiv a license to display the preprint in perpetuity. It is made available under aCC-BY 4.0 International license.

Supplementary Table 3. Primer sequences

	Sequence
IFNG (F)	TCAGCTCTGCATCGTTTTGG
IFNG (R)	TGGTCTCCACACTCTTTTGG
IL17 (F)	GCACAAACTCATCCATCCCC
IL17 (R)	TCCTCATTGCGGTGGAGATT
RPL19 (F)	GCGAGCTCTTTCCTTTCGCT
RPL19 (R)	TGCTGACGGGAGTTGGCATT
